# Alu-Mediated Insertions in the *DMD* Gene: A Difficult Puzzle to Interpret Clinically

**DOI:** 10.3390/ijms24119241

**Published:** 2023-05-25

**Authors:** Annalaura Torella, Alberto Budillon, Mariateresa Zanobio, Francesca Del Vecchio Blanco, Esther Picillo, Luisa Politano, Vincenzo Nigro, Giulio Piluso

**Affiliations:** 1 Department of Precision Medicine, University of Campania “Luigi Vanvitelli”, Via L. De Crecchio 7, 80138 Naples, Italy; annalaura.torella@unicampania.it (A.T.); alberto.budillon@hotmail.it (A.B.); mt.zanobio@gmail.com (M.Z.); francesca.delvecchioblanco@unicampania.it (F.D.V.B.); esther.picillo@unicampania.it (E.P.); vincenzo.nigro@unicampania.it (V.N.); 2 Telethon Institute of Genetics and Medicine, Via Campi Flegrei 34, 80078 Pozzuoli, Italy; 3 Department of Experimental Medicine, University of Campania “Luigi Vanvitelli”, Via L. De Crecchio 7, 80138 Napoli, Italy; luisa.politano@unicampania.it

**Keywords:** Becker muscular dystrophy, *DMD*, exon skipping, Alu-mediated transposon insertion, Log-PCR

## Abstract

Disrupting variants in the *DMD* gene are associated with Duchenne or Becker muscular dystrophy (DMD/BMD) or with hyperCKemia, all of which present very different degrees of clinical severity. The clinical phenotypes of these disorders could not be distinguished in infancy or early childhood. Accurate phenotype prediction based on DNA variants may therefore be required in addition to invasive tests, such as muscle biopsy. Transposon insertion is one of the rarest mutation types. Depending on their position and characteristics, transposon insertions may affect the quality and/or quantity of dystrophin mRNA, leading to unpredictable alterations in gene products. Here, we report the case of a three-year-old boy showing initial skeletal muscle involvement in whom we characterized a transposon insertion (Alu sequence) in exon 15 of the *DMD* gene. In similar cases, the generation of a null allele is predicted, resulting in a DMD phenotype. However, mRNA analysis of muscle biopsy tissue revealed skipping of exon 15, which restored the reading frame, thus predicting a milder phenotype. This case is similar to very few others already described in the literature. This case further enriches our knowledge of the mechanisms perturbing splicing and causing exon skipping in *DMD*, helping to properly guide clinical diagnosis.

## 1. Introduction

The conventional diagnosis of muscular diseases is often time-consuming and expensive due to the very large number of genes potentially involved in the presence of several, sometimes overlapping, manifestations. In addition, muscular diseases may be caused by single nucleotide variations (SNVs), copy number mutations (CNMs), or dynamic mutations. Achieving an accurate diagnosis often requires a multidisciplinary strategy combining clinical and molecular approaches. The *DMD* gene (MIM 300377) provides instructions for creating dystrophin protein. Pathogenic mutations affecting this gene cause a broad spectrum of X-linked dystrophinopathies, as different variants can lead to either the mild form of Becker muscular dystrophy (BMD; MIM 300376), the severe form of Duchenne muscular dystrophy (DMD; MIM 310200), or a form of X-linked dilated cardiomyopathy (XLCM; MIM 302045).

BMD is about one-third as frequent as DMD, with a newborn incidence of 1:11,500 in males [1]. BMD patients display variable and late-onset symptoms and have a longer life expectancy than children affected by DMD. Their gait may be conserved until adulthood or be waddling since childhood, and respiratory and cardiac muscles may also be involved during the progression of the disease, leading to respiratory distress or dilated cardiomyopathy [1,2,3]. As in other muscular diseases, both forms present elevated serum levels of creatine kinase (CK).

In the early stages, the distinction between BMD and DMD is not always clear. Therefore, early molecular findings should always be combined with a reading frame analysis of the DMD transcript, allowing the prediction of disease progression [4].

Transposons, also named transposable elements (TEs), are genetic elements that jump from one site of the genome to another. Their insertion is one of the rarest causes of altered gene expression. Since they are highly conserved in evolution, their genomic function is becoming increasingly recognized, and they make up a large portion of DNA in eukaryotic cells [5].

Alu repeats are the most frequent TEs, with more than one million copies in the human genome [6]. They are about 300 bp in length and belong to a class of retroelements called short interspersed nuclear elements (SINEs) [6]. Based on the target region of the insertion, they can either be silent or affect gene expression in different ways, producing mutations that are often challenging to detect.

Given that *DMD* is the largest human gene, it is unsurprising that some cases of transposon-induced dystrophinopathies have been described. Exonic and intronic TE insertions were reported to cause XLCM through exon skipping or exonization [7,8,9]. Conversely, only one patient was found to carry the insertion of an AluYa5 sequence in intron 56, leading to the skipping of exon 57 and a DMD phenotype [10].

We here expand the cohort of DMD/BMD patients with Alu-mediated causative variants, molecularly characterizing a three-year-old child with signs of skeletal muscle involvement, in whom we identified a transposon insertion (Alu sequence) in the *DMD* gene, leading to an aberrant skipping of exon 15 resulting in a Becker phenotype.

## 2. Results

### 2.1. Case Presentation

Here, we describe the complex case of a three-year-old boy with elevated serum CK levels and negative Gower’s sign. Because he was adopted at the age of two, no clinical data prior to that time and no family history are available.

The child’s clinical history began at 2 years old when he presented with painful seizures caused by vessel occlusion. Physicians at the University of Campania “Luigi Vanvitelli” Pediatric Hematology Unit suspected sickle cell anemia, subsequently confirmed by blood tests and hemoglobin electrophoresis. He was started on hydroxyurea, which brought about significant clinical improvement.

At the age of three, the child was found to have high serum CK levels (up to 35 times higher than the upper-limit normal levels) variable in the time and was referred to the University of Campania “Luigi Vanvitelli” Unit of Medical Genetics and Cardiomyology for a genetic evaluation. The boy was found to be mildly hypotonic, with no calf pseudohypertrophy. His gait was normal, he could easily walk up and down the stairs, and Gower’s sign was negative.

During the latest clinical evaluation at 9 years old, the patient did not show superior limb limitation to passive mobilization or difficulty in walking long distances or climbing stairs. He was also evaluated through The North Star Ambulatory Assessment (NSAA) with a global score of 34/34. CK levels settled down to an average 10 times higher than the normal upper level. His echocardiagraphic evaluation did not show specific alterations or signs of ventricle dilatation.

### 2.2. Molecular Diagnosis

Based on clinical evidence and high serum CK levels, two main diagnostic hypotheses were considered: an X-linked dystrophinopathy or an autosomal recessive limb-girdle muscular dystrophy (LGMD), given that the boy was born in a country with a high level of inbreeding. As the incidence of dystrophinopathies is significantly higher than that of LGMDs, we decided to investigate first for deletions/duplications in the *DMD* gene.

**Figure 1:**
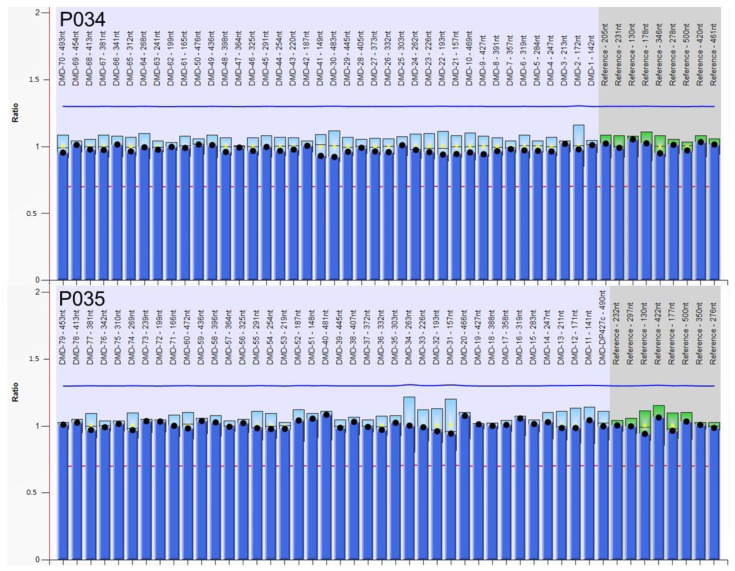
MLPA data analysis. Using the two *DMD*-specific MLPA probe mix (P034 and P035), the Coffalyser.net software detected no quantitative alterations in *DMD* exons from patient’s DNA (Dots correspond to the ratio value of each probe pair compared to control references).

We initially performed MLPA analysis, the gold standard for identifying CNMs in *DMD*. No deletions or duplications affecting one or more exons were detected (Figure 1).

**Figure 2:**
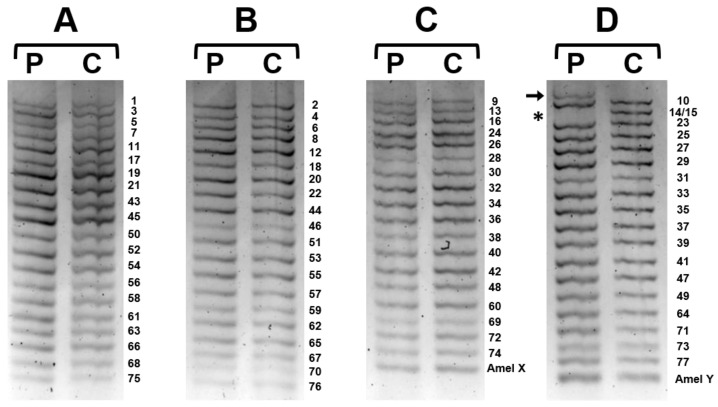
Log-PCR on patient (P) and normal control (C) samples. Electrophoresis of the four-primer mixes (**A**–**D**) including all *DMD* exons and AmelX/Y for sex determination. Mix D shows the absence of the band corresponding to exons 14 and 15 (*) as well as the presence of an additional upper band (arrow), not detected in the normal control.

Based on our internal workflow for DMD/BMD molecular diagnosis, we then performed Log-PCR [11], routinely used to confirm single exon deletions/duplications or as an alternative technique for excluding any event undetectable by MLPA. Interestingly, Log-PCR showed the absence of the band corresponding to exons 14 and 15 as well as the presence of an additional upper band, not detected in the negative control (Figure 2).

**Figure 3:**
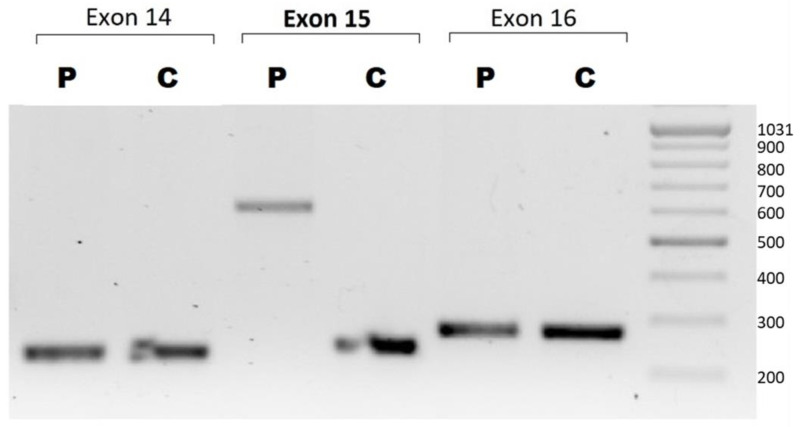
PCR of *DMD* exons 14, 15, and 16. Qualitative analysis of *DMD* exon 15 on agarose gel revealed that the band of the patient (P) was higher than that of the control (C), suggesting the presence of an insertion.

**Figure 4:**
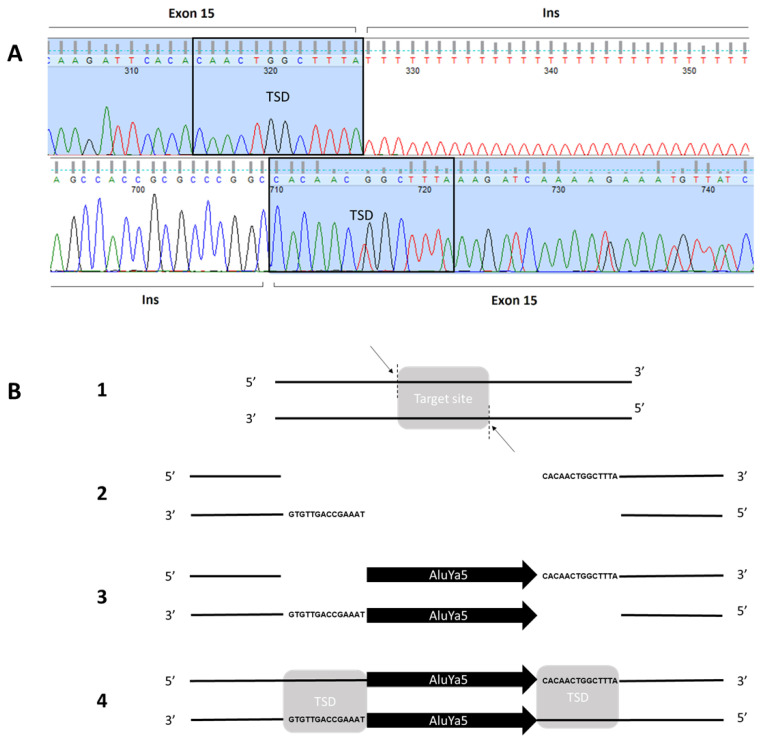
Panel (**A**). Sequence of genomic *DMD* exon 15 (electropherogram reports the peaks corresponding to the different nucleotides in different colors corresponding to sequence of bases on the top). Sequence of *DMD* exon 15 is in light blue and it holds target site duplications (TSD) produced by the insertion. Nonhighlighted nucleotides are, respectively, the ending and the starting part of transposon element (AluYa5). Panel (**B**). Scheme for AluYa5 insertion in Exon 15. (1) Trasposase selects and (2) cleaves target DNA on two single strands. (3) AluYa5 inserts in the gap. (4) Polymerase fills the vacuity forming two target site duplication (TSD) at the edges.

In order to explain the discrepancy between the findings obtained using these two methods, we performed a PCR analysis of individual exons 14, 15, and 16, which revealed that the size of the exon 15 band did not match that of the control band (Figure 3). Sanger sequencing of exon 15 identified an antisense-oriented TE corresponding to an Alu sequence [NM_004006.2:c.1771_1772ins353] (Figure 4). Based on a search for repetitive DNA elements in the Dfam database (Dfam 2.0 software, University of Montana, MT, USA), our sequence completely matched the consensus sequence of the AluYa5 subtype element.

**Figure 5:**
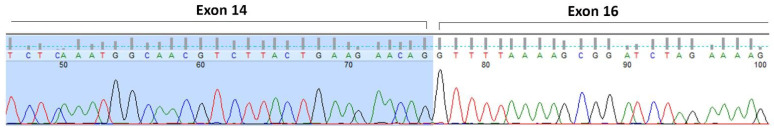
Exon 14–16 cDNA sequence. In line with exon skipping of exon 15, the cDNA sequence shows exons 14–16 junction in the patient’s *DMD* transcript.

To further investigate the functional effects of this insertion, we performed a muscle biopsy and analyzed the dystrophin cDNA. The amplification of a fragment corresponding to exons 14–16 and its subsequent sequencing showed the lack of exon 15 in the analyzed transcript, thus indicating a partially functioning in-frame transcript produced by exon 15 skipping (Figure 5).

Our analysis identified a rare variant in *DMD* causing muscular dystrophy in the patient. No similar mutation has previously been described in the literature, and because the boy was adopted, we were unable to know of any other potential cases in his biological family. At the time of investigation, a BMD phenotype was hypothesized for the patient, mainly based on cDNA analysis, which showed an in-frame *DMD* transcript, and on the Monaco rule [12].

## 3. Discussion

Here, we describe a patient with a rare and previously unreported molecular variation in the *DMD* gene.

Based on the mutation type in *DMD*, the diagnostic workflow should first investigate CNMs (which constitute around 75% of causative variants) by MLPA and/or Log-PCR, and subsequently explore SNVs using next-generation sequencing and/or Sanger sequencing [13].

Our experience in neuromuscular diseases has led us to the conclusion that, due to the complexity of some elusive mutations, the use of independent methods is always worthwhile, and that a combination of multidisciplinary clinical and molecular skills is often required to solve the most challenging cases.

MLPA is currently the gold standard technique for detecting CNMs causing DMD/BMD. In our patient, MLPA was not able to obtain a diagnosis as the hybridization of the exon 15-specific *DMD* probe pair was not perturbed by the Alu element insertion. Conversely, Log-PCR revealed a shift of the band corresponding to exons 14 and 15 (amplified together as they are less than 200 bp apart) resulting from the Alu element insertion, which moved its position higher in the gel. Sanger sequencing confirmed an insertion of 353 nucleotides in exon 15 of *DMD*, corresponding to the AluYa5 sequence and resulting in the in-frame skipping of the exon in the *DMD* muscular isoform. This finding was further confirmed in a separate study investigating the effectiveness of single-molecule real-time sequencing technology (PacBio, Menlo Park, CA, USA) in detecting splicing defects that we had identified using other independent methods [14].

Although MLPA is a standard and widely used protocol, the case presented here strongly supports the combined use of alternative and independent techniques to detect uncommon causative variations. Log-PCR, for example, is routinely performed in our laboratory workflow for DMD/BMD genetic testing.

Molecular diagnosis can greatly assist clinicians in differentiating severe (DMD) and mild (BMD) forms of dystrophinopathy, which have a dramatically different impact on quality of life and life expectancy. The Monaco rule [12] and cDNA analysis confirmed that the identified mutation in our patient produced a partially functioning in-frame *DMD* transcript through the skipping of exon 15, presumptively leading to a long-term mild phenotype comparable to BMD. To date, no similar cases have been reported in the literature, although Wang et al. described a BMD patient with a splice site variant (NM_004006.2:c.1812 + 1G > A) likely inducing the skipping of exon 15 in *DMD* [15]. In addition, nonsense mutations in the dystrophin gene can, in some cases, be rescued by in-frame exon skipping, carrying a milder BMD phenotype [4]. A similar case with the concurrent identification of a very mild myopathic phenotype caused by DMD exon 15 skipping in a patient with Down syndrome has been recently reported [16]. Immunohistochemistry of dystrophin showed the correct dystrophin localization at the sarcolemma, with only weaker immunostaining with rod-domain specific antibodies. The clinical presentation of this patient perfectly overlaps with that observed in our patient. Considering all these findings, our analysis indicates a diagnosis of BMD, which is supported by the follow-up of the patient, who still presents very mild muscle signs with no appreciable worsening to date.

The mechanism underlying the insertion of an Alu sequence and the subsequent skipping of *DMD* exon 15 is still poorly understood. Nakama et al. reported that an intronic antisense Alu element had a negative splicing effect on the inclusion of the adjacent downstream exon of *ACAT1*. The authors also found that the same effect is not observed through a sense insertion and that the distance between the Alu element and the skipped exon is fundamental to negatively affecting its inclusion in the transcript [17]. Using a minigene assay approach, another study showed that a 16-bp sequence in the Alu element played a key role in exon skipping and could provide a crucial binding site for splicing factors controlling exon skipping. The authors also speculate that the antisense-oriented Alu may shape a double-stranded pattern [18]. An Alu intronic insertion was also described by Lev-Maor et al. for *RABL5*, determining the splicing of the flanking exon [19].

**Figure 6:**
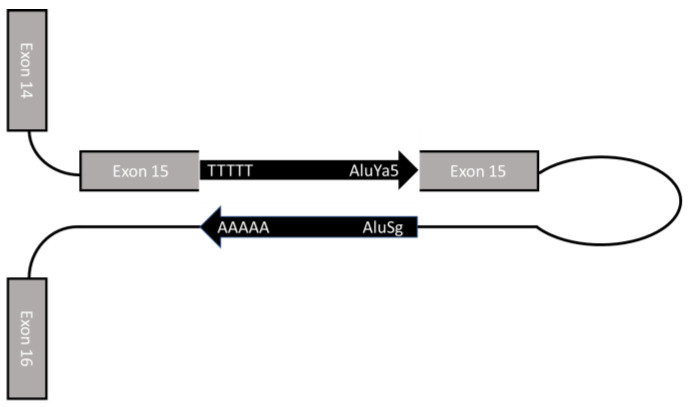
Hypothetical folding of the primary transcript. The insertion of the AluYa5 element in the exon 15 might form a double-stranded secondary structure with the AluSg physiologically present in the opposite orientation in the intron 15 (negative strand).

A similar genomic structure was found in the genomic region containing exon 15 of *DMD*. In fact, an Alu element (AluSg) is located in intron 15, in the opposite orientation compared to the *DMD* gene. Furthermore, since intron 14 is only 159 bp in length, *DMD* exons 14 and 15 are very close (Figure 6).

All these findings prompted us to speculate the involvement of a mechanism similar to that proposed by Nakama et al. and Lev-Maor et al. [17,18,19]. The insertion of AluYa5 in exon 15 might form a double-stranded secondary structure with the AluSg physiologically present in the opposite orientation in intron 15. This could likely impair the binding of the splicing factors, thus negatively affecting exon 15 inclusion in the transcript.

## 4. Materials and Methods

### 4.1. Multiplex Ligation-Dependent Probe Amplification

A total of 50 ng of genomic DNA extracted from whole blood cells was used to perform a multiplex ligation-dependent probe amplification (MLPA) assay with a SALSA MLPA P034/P035 DMD kit (MRC Holland, Amsterdam, The Netherlands), according to the manufacturer’s instructions. MLPA data analysis was carried out using the Coffalyser.net software ver. 220513.1739 (MRC Holland, Amsterdam, The Netherlands).

### 4.2. Log-PCR

The patient’s genomic DNA (60–100 ng) was used to amplify all *DMD* exons and flanking introns by performing multiplex and semiquantitative PCR, as previously described [11].

### 4.3. Polymerase Chain Reaction and Sanger Sequencing

The genomic region corresponding to exons 14, 15, and 16 of *DMD* was amplified by PCR using the patient’s genomic DNA. The altered fragment in the patient, corresponding to exon 15, was then directly sequenced using specific primers and a BigDye version 3.1 sequencing kit (Applied Biosystems, Waltham, MA, USA) on a 3500xL Genetic Analyzer (Applied Biosystems, Waltham, MA, USA), according to the manufacturer’s instructions.

### 4.4. RT-PCR

After the local administration of 2% lidocaine, a skin biopsy was performed in the perioral region using a 2 mm punch, as previously reported [20]. The skin sample was rich in dermal annexes and totally used for RNA extraction. Total RNA was obtained from the patient’s biopsy using TRIzol RNA isolation reagents (Thermofisher Scientific, Waltham, MA, USA), according to the manufacturer’s specifications. RNA was then retrotranscribed using SuperScript III RT (Invitrogen, Carlsbad, CA, USA) and random primers, according to the manufacturer’s instructions. Complementary DNA (cDNA) was then used to amplify *DMD* fragment spanning exons 14–16. The PCR product was subsequently analyzed by bidirectional sequencing, as previously described (see Sanger sequencing section).

## 5. Conclusions

By combining multiple techniques, we solved a complex molecular case by identifying an elusive mutation in the *DMD* gene. These Alu-mediated pathogenic mechanisms are still poorly understood and rarely detected, particularly in a gene as big as DMD. Our hypothesis is that the Alu insertion in *DMD* exon 15 may affect physiological splicing if combined with the presence of a similar antisense-oriented sequence nearby. Our findings provide an additional model of transposon-mediated alteration of gene expression.

## Data Availability

Data are contained within the article.
